# Estimating changes in metabolic power from EMG

**DOI:** 10.1186/2193-1801-2-229

**Published:** 2013-05-19

**Authors:** Ollie M Blake, James M Wakeling

**Affiliations:** Department of Biomedical Physiology and Kinesiology, Simon Fraser University, 8888 University Drive, Burnaby, British Columbia V5A1S6 Canada

**Keywords:** Electromyography, Oxygen uptake, Cycling, Muscle

## Abstract

Metabolic rates can increase 21 times above resting levels during cycling with the majority attributed to muscular contractions. Metabolic estimates attained through gas exchange parameters are limited by the respiration rate and time delay with respect to these contractions. In contrast surface electromyography (EMG) contains instantaneous muscle contraction information at higher temporal resolutions. An adequate metabolic power-EMG relationship has not been established to use EMG as a metabolic estimate during dynamic activities. The purpose of this study was to establish a metabolic power-EMG relationship during non steady-state conditions. Participants cycled at workloads between 25 and 90% O_2max_ while EMG and gas exchange were monitored. The EMG was resolved into intensities and total EMG intensity was calculated as the sum of intensities across all muscles for each pedal cycle. Metabolic power was estimated from gas exchange parameters and the mean total EMG intensity between breaths was calculated and used as breath-by-breath values. Comparisons were made between breath-by-breath resolutions of metabolic power and total EMG intensity. Different weighting coefficients were also applied to the EMG for each muscle to analyze the effects of different muscle weightings on metabolic power estimations. There was a significant correlation (r = 0.91) between estimates of metabolic power from EMG and gas exchange. Muscle weighting had a significant effect on metabolic power determination with the highest and lowest correlated estimates having the largest weightings on muscles proximal and distal to the knee respectively. This study demonstrates that EMG contains important information about the metabolic costs of muscle contractions and provides good predictions of metabolic changes during non steady-state conditions. Also, the importance of each muscle is workload dependent with inappropriate weightings reducing metabolic estimations. These findings have implications for future EMG applications as they provide more immediate, higher temporal resolution predictions of changes in metabolic power.

## Background

Contracting muscles require energy and more energy is needed as muscular force production increases. Metabolic rates can increase 21 times above resting levels in trained cyclists (Astrand & Rodahl 1986), which is primarily attributed to the energy supplied to the contracting leg muscles by aerobic and anaerobic sources. Muscle contractions during dynamic activities must therefore contain considerable information about metabolic power.

Aerobic metabolism during isometric or dynamic activities is often estimated through indirect calorimetry using oxygen and carbon dioxide gas exchange where the energy utilized by the working muscles reflects the changes in pulmonary oxygen uptake (Poole et al. 1992). Unfortunately measures of metabolic power based on gas exchange are unable to resolve metabolic costs to a resolution greater than the respiration rate. Consequently, information about muscular contractions that influence metabolic power is neglected since these contractions can occur more frequently than the respiration rate.

Changes in force during muscular contractions are primarily achieved by altering the number of active motor units and the motor unit firing rates. These changes can be detected using surface electromyography (EMG), which provides information about the active muscle by measuring the electrical signals of the motor unit action potentials. Gait cycles can take less than a second during high-speed movements and EMG fluctuates considerably within this time. EMG therefore must contain information about metabolic power at a higher temporal resolution than gas exchange measures, yet it is unknown if EMG can be used to estimate metabolic power changes during non steady-state dynamic activities.

Previous studies looking at the relationship between oxygen uptake ( O_2_) and EMG have shown evidence of a linear relationship below the anaerobic threshold (Arnaud et al. 1997;Bigland-Ritchie & Woods 1976;Jammes et al. 1998) and a non-linear relationship above the anaerobic threshold (Hug, Decherchi, et al. 2004a). The non-linearity of this relationship is partially explained by a greater increase in EMG than O_2_ due to a larger reliance on anaerobic metabolism. We recently found a significant monotonic increase in metabolic power (r = 0.86), estimated from O_2_ that was associated with increased EMG intensity (Wakeling et al. 2011). Mean values of O_2_ and EMG intensity were calculated while cycling at or near steady-state at a range of workloads from 25-90% maximum oxygen uptake ( O_2max_). Although this relationship was significant it only indicates that metabolic power is related to EMG intensity during steady-state activity when averaged over an extended time. The relationships at the workload transitions were not investigated and the higher temporal resolution of the EMG was ignored. Dynamic activities are rarely performed at constant workloads except during controlled experiments. It is therefore important to assess the relationship between metabolic power and EMG in non steady-state conditions.

Oxygen uptake kinetics in response to a stepwise change in workload can be modeled as exponential processes distinguished by changes in arterial and venous blood oxygen content (Jones & Poole 2005). This provides good predictions of the oxygen uptake kinetics underlying the breath-by-breath fluctuations. Adequate changes in cardiac output and O_2_ to accommodate oxygen demands of the working muscles are delayed compared to the EMG signal since the altered venous blood oxygen content takes time to reach the lungs. For example, Whipp and co-workers (1982) showed that, in response to a stepwise increase in workload, there exists a time delay of approximately 20 seconds accounting for approximately 20% of the total increase in O_2_ before the primary exponential rise towards steady-state.

The purpose of this study was to further define metabolic changes based on EMG by establishing a significant relationship between metabolic power and EMG on a breath-by-breath basis in non steady-state conditions. It was hypothesized that a significant relationship exists at a greater temporal resolution than previously determined and good estimates of changes in metabolic power would be established from the EMG signal.

## Results

**Figure 1:**
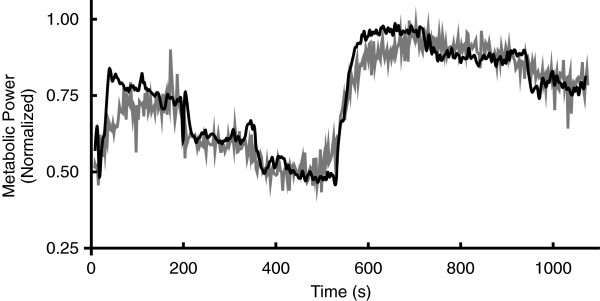
**Estimates of metabolic power.** Metabolic power (grey) calculated from oxygen uptake and estimated metabolic power (black), calculated from the EMG signal using the transfer function (Eq. 1), for one participant (subject 6). The transfer function was optimized with a time delay of 23.65 s, β of 0.25 and τ of 87 breaths resulting in a correlation of r = 0.94.

The correlation between breath-by-breath temporal resolutions of EMG intensity and metabolic power was r = 0.80 ± 0.02, which improved to r = 0.85 ± 0.02 when accounting for the time delay through cross-correlation. There was a significant correlation (r = 0.91 ± 0.01) between metabolic power determined from the EMG intensity using the transfer functions (Eq. 1) and the metabolic power estimated from O_2_ (Table 1; Figure 1). From the transfer function, the mean time delay (*t*_delay_) between the EMG signal and the subsequent metabolic power was approximately 12 breaths, which equates to 28.33 ± 4.31 s, with a mean β of 0.28 ± 0.07 and τ of 96.11 ± 2.57 breaths (Table 1).

**Table 1:** **Time delay, transfer function coefficients (**τ **and β) and correlations for each subject to estimate metabolic power from EMG**

Subject	Time delay	τ	β	Correlation
1	34.56	100	0.30	0.88
2	16.90	100	0.10	0.88
3	13.20	99	0.20	0.87
4	17.32	79	0.10	0.87
5	48.12	100	0.35	0.93
6	23.65	87	0.25	0.94
7	20.55	100	0.70	0.92
8	34.93	100	0.45	0.94
9	45.72	100	0.05	0.95
**Mean**	28.33	96.11	0.28	0.91
**SEM**	4.31	2.57	0.07	0.01

The time delay is the time adjustment from the measurement of the EMG signal to the subsequent change in gas exchange parameters calculated from the transfer function.

**Figure 2:**
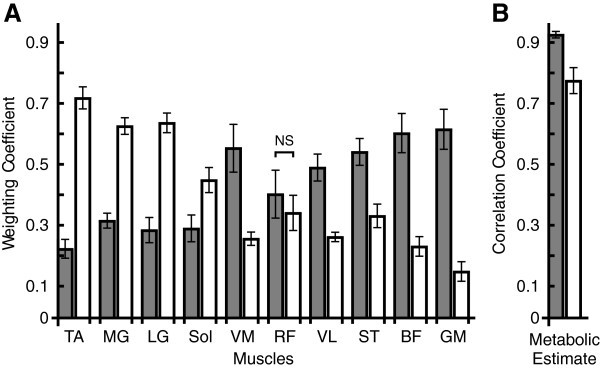
**Muscle weighting coefficients and resultant metabolic estimates correlated with the metabolic estimates from****O**_**2**_**.** (**A**) Mean ± SEM weighting coefficients for each muscle for the highest (H_cor_; grey) and lowest (L_cor_; white) 100 correlated estimates of metabolic power. (**B**) Mean ± SEM correlations between metabolic estimates from O_2_ and both H_cor_ (r = 0.93 ± 0.01) and L_cor_ (r = 0.77 ± 0.04). The correlation and weighting coefficients for H_cor_ and L_cor_ for each muscle were significantly different except for rectus femoris (RF) as indicated (NS).

Applying different weights to each muscle in the model had a significant effect on the determination of metabolic power as seen by the significantly different mean correlations for the 100 highest (Hcor; see Methods) and 100 lowest (Lcor) correlated muscle weighting combinations (r = 0.93 ± 0.01 and r = 0.77 ± 0.04 respectively; Figure 2b). H_cor_ had the highest mean weightings for vastus medialis (VM; 0.55 + 0.08; Figure 2a), vastus lateralis (VL; 0.49 ± 0.04), semitendinosus (ST; 0.54 ± 0.04), biceps femoris (BF; 0.60 ± 0.07) and gluteus maximus (GM; 0.62 ± 0.07), while L_cor_ had the highest mean weightings for tibialis anterior (TA; 0.72 ± 0.04; Figure 2a), medial gastrocnemius (MG; 0.63 ± 0.03) and lateral gastrocnemius (LG; 0.63 ± 0.03). Significantly greater weightings were placed on VM, VL, ST, BF and GM for H_cor_ than for L_cor_ and significantly greater weightings were placed on TA, MG, LG and soleus (Sol) for L_cor_ compared to H_cor_. The weighting for rectus femoris (RF) was not significantly different between H_cor_ and L_cor_.

**Figure 3:**
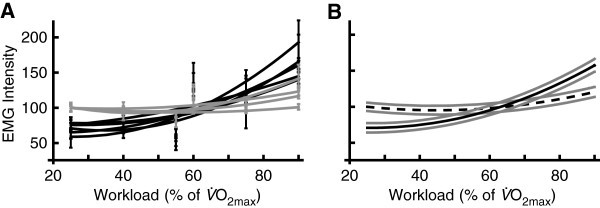
**Mean ± SEM EMG intensity for each workload between 25 and 90%****O**_**2max**_**.** (**A**) Muscles distal to the knee (TA, MG, LG, Sol; grey lines) show relatively little change in EMG intensity across workloads and muscles proximal to the knee (VM, RF, VL, ST, BF and GM; black lines) show relatively large increases in EMG intensity at workloads above 55% O_2max_. (**B**) Mean (black lines) ± SEM (grey lines) across all muscles with muscle bellies distal (dashed line) and proximal (solid line) to the knee.

The muscles with muscle bellies distal to the knee (TA, MG, LG and Sol) displayed less range in EMG intensity across workloads than those proximal to the knee (VM, RF, VL, ST, BF and GM; Figure 3). The muscles proximal to the knee showed little change in EMG intensity below 60% O_2max_ and large increases above 60% O_2max_. In most muscles EMG intensity for the 60% O_2max_ condition did not follow the best-fit curve and was significantly higher than the 55% O_2max_ condition.

## Discussion

The primary finding of this study was the significant correlation (r = 0.91) between estimates of metabolic power from gas exchange and EMG intensity at a higher temporal resolution than the previously established relationship between metabolic power and EMG intensity (r = 0.86; (Wakeling et al. 2011)). This was important as it included workload transitions as found in non steady-state conditions. This indicates that breath-by-breath EMG intensities provide a good estimate of changes in metabolic power.

The time-shifted correlation between metabolic power and EMG intensity (r = 0.85) revealed an improved relationship over the initial breath-by-breath correlation (r = 0.80). The *t*_delay_ from the measured EMG signal to the subsequent O_2_ realized at the mouth was approximately 12 breaths or 28.33 ± 4.31 seconds. This is reasonable given the time delay of approximately 20 seconds attributed to the delay in the transport of blood from the working muscles to the lungs (Whipp et al. 1982). The EMG signal therefore contains metabolic information closer in time to the actual energy use than gas exchange since the *t*_delay_ is only apparent because the estimated metabolic power was measured at the mouth.

**Figure 4:**
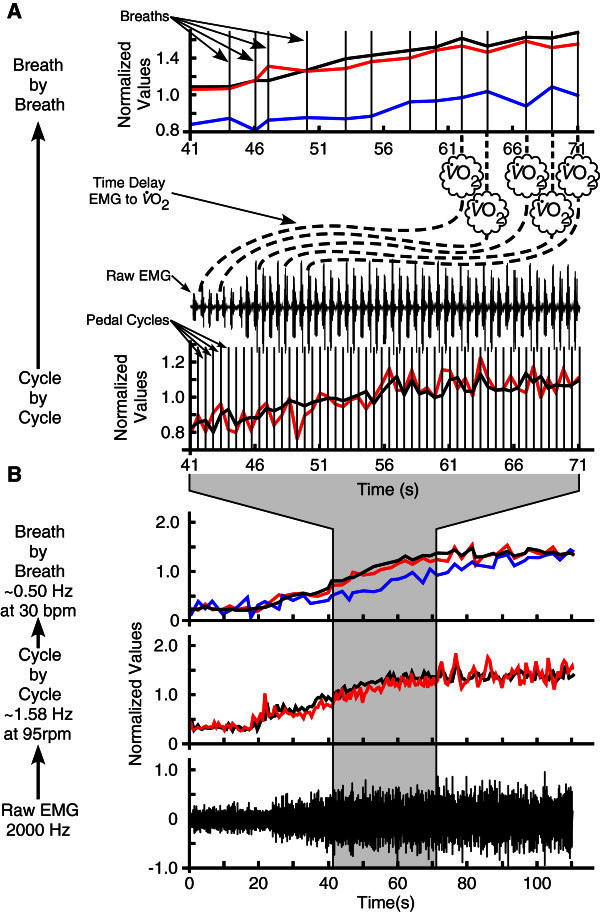
**Cycle-by-cycle and breath-by-breath temporal resolutions of the EMG and volume of oxygen uptake signals.** (**A**) Visual representation of 30 seconds of data during a change in workload (power output; solid black line) and the total EMG intensity (EMG; red line) and volume of oxygen uptake ( O_2_; blue line) responses. The cycle-by-cycle figure shows the individual pedal cycles (vertical lines) and the cycle-by-cycle variation in the EMG that is matched well with variation in power output at this temporal resolution. Moving from cycle-by-cycle to the breath-by-breath temporal resolution (mean EMG and power output between breaths) the EMG and power output curves are smoothed since there were 3.19 ± 0.27 pedal cycles per breath across all workloads. Also shown is the time delay (dashed lines) of 28.33 ± 4.31 seconds between a change in the EMG signal and power output and the subsequent change in O_2_. (**B**) A global perspective over 110 seconds moving from raw EMG sampled at 2000 Hz to cycle-by-cycle temporal resolution at approximately 1.58 Hz at 95 pedal revolutions per minute (rpm) to breath-by-breath temporal resolution at approximately 0.50 Hz at 30 breaths per minute (bpm). This clearly shows the cycle-by-cycle variation of the EMG and power output that is reduced moving to breath-by-breath resolution, while the primary features of the underlying kinetics are preserved. The processed EMG, power output and O_2_ values have been normalized to the mean across the 110 seconds of data, while the raw EMG is normalized to the absolute maximum.

Due to the breath-by-breath fluctuations in both O_2_ and EMG (Figure 4), the activation constants of the transfer functions do not possess significant physiological information. Instead these optimized values smooth the metabolic estimate and highlight the underlying kinetics in order to maximize the correlation. This has a similar effect to previous methods for modeling oxygen uptake using exponential functions that highlight the underlying kinetics of the breath-by-breath fluctuations (Jones & Poole 2005). There is no physiological explanation to indicate that these fluctuations are directly related to the contractions of the individual muscles, and therefore evident in the EMG signal, since they represent ‘white noise’ and are not important to the overall gas exchange kinetics (Lamarra et al. 1987) and metabolic estimations. This implies that the fundamental information about metabolic power is contained in the EMG signal and the transfer function (Eq. 1) operates to emphasize the essential features despite the unrelated noise.

The maximum allowable value of τ in the transfer function (Eq. 1) was 100 breaths, yet six subjects had optimized values at this maximum (Table 1). Values greater than 100 breaths were also tested to ensure the resultant correlations were maximized. No subjects displayed appreciable gains in correlation (no difference in r values to the four significant digits reported) with values of τ above 100 breaths. The aim of this study was not to identify specific transfer function constants, but to show a significant relationship between metabolic power estimated from gas exchange and EMG. This was accomplished within the constraints imposed on the constants.

There were 3.19 ± 0.27 pedal cycles per breath across all workloads, which provides a small measure of smoothing, similar to a moving average, for the breath-by-breath EMG intensities when compared to the cycle-by-cycle values. Even at 25% O_2max_, with the lowest mean respiration rate of 23.83 ± 1.75 breaths per minute, there were only 4.15 ± 0.36 pedal cycles per breath. This level of data reduction maintains the important information of the EMG signal while reducing the amount of noise (Figure 4). Determination of an instantaneous measure of metabolic cost remains elusive, but these data and the small number of pedal cycles between breathes suggests that cycle-by-cycle EMG intensity with a time resolution of approximately 0.70 seconds would also provide a reasonable estimate of the O_2_ kinetics and therefore the metabolic power.

Similar to previous findings (Ericson 1986;Hug & Dorel 2009;Hug, Bendahan, et al. 2004b;Lawrence & De Luca 1983), some muscles displayed non-linear relationships between the EMG signal and force or workload (Figure 3). This shows that the importance of each muscle is workload dependent. Different weightings were applied to each muscle in order to evaluate its significance to the estimation of metabolic power. Given the importance of the larger knee extensor muscles in cycling (Ericson 1986;Hug & Dorel 2009), it was predicted that a reduced muscle set would provide as accurate predictions of metabolic changes as the entire set of muscles. In fact combinations of all muscles resulted in the best estimates and inappropriate weighting resulted in significantly reduced estimations (Figure 2). These results provide insight into the most important muscles for accurate metabolic predictions and imply that each muscle has a different role in metabolic power depending on the relative workload intensity. Therefore, when cycling involves a range of workloads, as is the case in this study, it is particularly important to include EMG from many muscles to attain an accurate account of metabolic power.

Muscles proximal to the knee, which include the muscles considered to be the primary power producers in cycling (VL and VM, (Ericson 1986)), were more heavily weighted in H_cor_. Eliminating muscles distal to the knee with the lowest weights (TA and Sol) did not improve the metabolic estimations as these muscles had high relative EMG intensities at lower workloads (Figure 3). The EMG intensities for the 60% O_2max_ condition are inflated for all muscles (Figure 3) since they occurred after the 75% or 90% conditions for some participants. The 75% and 90% conditions occurred with respiration exchange ratios greater than one (Blake et al. 2012) indicating a greater contribution of anaerobic energy sources. With the 60% condition taking place after these workloads and no rest between conditions, there was likely increased fatigue, which is associated with higher levels of EMG intensity (Edwards & Lippold 1956;Petrofsky 1979). These inflated values decrease the curvature of the best-fit relationship of EMG intensity across all workloads, especially for those muscles proximal to the knee (Figure 3). Despite these limitations the EMG signals contained substantial information about the metabolic power as evidenced by the significant correlation between the metabolic power estimations (r = 0.91).

Normalization of the EMG signals to the mean across all trials for each subject and each muscle presents a limitation to the interpretation of the EMG-metabolic cost relationship. This method weights all muscles the same and ignores metabolic differences of the structural and contractile properties of the individual muscles. Interestingly, imposing 5000 different weighting combinations to the muscle EMG signals only increased the metabolic power estimation correlation from r = 0.91 to r = 0.93 (H_cor_). Changing the weightings is equivalent to using different normalization values for each muscle, and so the 5000 weighting combinations represented 5000 ways to normalize the muscles. The normalization method chosen for this study resulted in close to the best possible normalization for predicting metabolic power from EMG for this cycling task. However, an inappropriate set of normalization could result in poor results as shown by the significantly reduced correlation of L_cor_ (r = 0.77; Figure 2).

## Conclusions

This study shows that breath-by-breath changes in EMG across ten leg muscles can be used as a good estimate of changes in metabolic power in non steady-state conditions. Estimations of metabolic changes on a cycle-by-cycle basis may also be reasonable given that breath-by-breath resolution acts as a moving average of approximately three to four pedal cycles, thereby maintaining the important features of the data. This has implications for future studies and applications involving EMG from large muscle groups during dynamic activities as it provides higher temporal resolution and more real-time predictions of changes in metabolic power than gas exchange measures. Also, metabolic power estimated from gas exchange is used in calculations of mechanical efficiency, which implies that breath-by-breath changes in EMG can also be used to investigate the relationship of mechanical work and metabolic power.

## Methods

Nine competitive male cyclists (age = 41.8 ± 2.7 yr, mass = 77.2 ± 2.2 kg, height = 1.81 ± 0.01 m, O_2max_ = 64.65 mL kg^**-**1^ min^**-**1^ , yearly mileage = 9428 ± 1913 km; mean ± SEM) participated in the study. The participants gave their informed written consent, and the ethics committee in accordance with the Office of Research Ethics at Simon Fraser University approved all procedures.

The cycling protocol and data collection was completed as described previously (Blake et al. 2012). Briefly, participants cycled at a freely chosen cadence (94.7 ± 1.1 rpm) continuously for 18 minutes in three minute intervals at 25, 40 and 55% of the power output at O_2max_ in random order followed by 60, 75 and 90% O_2max_, also in random order, while breath-by-breath oxygen and carbon dioxide gas exchange (Vmax 229 metabolic cart, Sensormedics, Yorba Linda, California) and EMG from 10 leg muscles (tibialis anterior (TA, MG, LG, Sol, VM, RF, VL, ST, BF and GM)) were recorded. The EMG recording was completed using bipolar Ag/AgCl surface electrodes (10mm diameter, 21mm spacing) and was amplified (factor of 1000), band-pass filtered (bandwidth 10-500 Hz; Biovision, Wehrheim, Germany) and recorded at 2000 Hz through a 16-bit A/D converter (USB-6210, National Instruments, Austin, TX). The EMG was resolved into intensities using wavelets (Von Tscharner 2000) and normalized to the mean intensity for each participant for each muscle across all conditions. The EMG intensity is a measure of the power of the EMG signal in the frequency range 10-500 Hz. Total EMG intensity was calculated as the sum of the EMG intensities from each muscle for each pedal cycle and thus was termed the cycle-by-cycle value (Figure 4).

Breath-by-breath total EMG intensities were determined by taking the mean total EMG intensity of those pedal cycles between each breath (Figure 4). Pedal cycles containing breath measurements were included in the following breath. Metabolic power was calculated from the gas exchange parameters using caloric equivalents of oxygen (Foss et al. 1998). For each subject Pearson correlation coefficients were calculated between the breath-by-breath measures of EMG intensity and metabolic power. Also cross-correlation was used to determine the correlation coefficient when accounting for the time delay between the onset of EMG and subsequent gas exchange measure of metabolic power. Metabolic power (*P* in Eq. 1) was also estimated from the EMG intensity using a bilinear differential equation (Zajac 1989) that has been used previously to determine the active state of a muscle from its EMG excitation (Lee et al. 2011). The transfer functions accounted for and were defined by the time delay (*t*_delay_), time constant of rise in metabolic power (τ) and ratio of rise and decay of metabolic power (β). These constants were determined such that they maximized the correlation between estimations of metabolic power from gas exchange and EMG intensity subject to the following constraints: 0 ≤ τ ≤ 100 breaths, 0 ≤ β ≤ 2 and 0 ≤ *t*_delay_ ≤ 100 breaths.1

Variation in the structural and contractile properties of different muscles may be reflected in energy requirement differences not identified in the EMG signals since each muscle is normalized to its own mean for each subject across all trials. Therefore, in a subsequent analysis, different weighting coefficients were applied to the EMG intensity from each muscle to analyze the effect of muscle weighting on the correlation between the estimations of metabolic power from the EMG and gas exchange. Random weightings were assigned to the individual muscles in 5000 different combinations for each subject. These were then used in the transfer function, re-optimizing for the coefficients (τ, β and *t*_delay_), to predict metabolic power and re-calculate the estimated metabolic power correlations. In order to evaluate the differences in the individual muscle weighting coefficients that produced the best and worst metabolic power correlations, mean weightings of the 100 highest (H_cor_) and lowest (L_cor_) correlated muscle combinations were compared. Two-sided t-tests were conducted between H_cor_ and L_cor_ weighting coefficients for each muscle to determine if the mean values were significantly different. Statistical tests were considered significant at p ≤ 0.05 and results are reported as mean ± SEM.
